# A Hidden Markov Model for identifying essential and growth-defect regions in bacterial genomes from transposon insertion sequencing data

**DOI:** 10.1186/1471-2105-14-303

**Published:** 2013-10-08

**Authors:** Michael A DeJesus, Thomas R Ioerger

**Affiliations:** 1 Department of Computer Science, Texas A&M University, College Station, TX 77843, USA

**Keywords:** Next-generation sequencing, Sequence analysis, Hidden Markov Models, Essentiality

## Abstract

**Background:**

Knowledge of which genes are essential to the survival of an organism is critical to understanding the function of genes, and for the identification of potential drug targets for antimicrobial treatment. Previous statistical methods for assessing essentiality based on sequencing of tranposon libraries have usually limited their assessment to strict 'essential’ or 'non-essential’ categories. However, this binary view of essentiality does not accurately represent the more nuanced ways the growth of an organism might be affected by the disruption of its genes. In addition, these methods often limit their analysis to open-reading frames. We propose a novel method for analyzing sequence data from transposon mutant libraries using a Hidden Markov Model (HMM), along with formulas to adapt the parameters of the model to different datasets for robustness. This approach allows for the clustering of insertion sites into distinct regions of essentiality across the entire genome in a statistically rigorous manner, while also allowing for the detection of growth-defect and growth-advantage regions.

**Results:**

We evaluate the performance of a 4-state HMM on a sequence dataset of *M. tuberculosis* transposon mutants. We also test the HMM on several synthetic datasets representing different levels of transposon insertion density and sequence coverage. We show that the HMM produces results that are highly correlated with previous assignments of essentiality for this organism. We also show that it detects growth-defect and growth-advantage genes previously shown to impair or enhance growth when disrupted.

**Conclusions:**

A 4-state HMM provides an improved way of analyzing Tn-seq data and assessing different levels of essentiality that enables not only the characterization of essential and non-essential genes, but also genes whose disruption leads to impairment (or enhancement) of growth.

## Background

**Figure 1:**
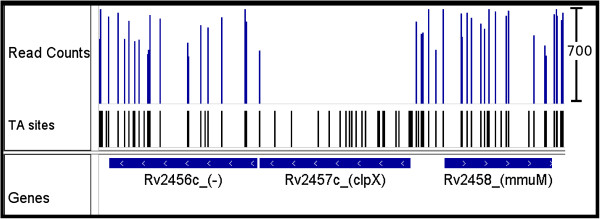
**ClpX read counts.** Example read counts for a 4kb region in the *M. tuberculosis* genome obtained from deep-sequencing of transposon insertion libraries. ClpX, the ATP-dependent specificity component of the CLP protease, contains a single insertion in the C-terminus. The 19 remaining TA sites are devoid of insertions, indicative of an essential gene. Figure created using IGV - distributed by the Broad Inst. http://www.broadinstitute.org/igv/.

Transposon mutagenesis is an experimental method frequently used for surveying bacterial genomes for essential regions, including genes, as well as individual protein domains, regulatory elements, and non-coding RNAs that are required for survival. For example, the Himar1 transposon inserts randomly into TA nucleotides [1]. Those locations that lack a transposon insertion suggest either that the location is essential (as they could not tolerate disruption) or that it was not represented in the library of transposon mutants (i.e. the location is non-essential but transposons missed this location during construction of the library, resulting in incomplete saturation). Typically, around 15% of the genes in the genome of prokaryotic organisms are essential [2]. Knowledge of which genes are essential can be very useful for drug discovery against pathogens (e.g. to identify new targets for antibiotics). While the original method used hybridization on DNA microarrays [3,4], deep sequencing has made analysis of transposon insertion libraries much more efficient (sometimes called Tn-Seq or TRACS) [5,6]. Short reads are obtained from the genome on either side of each transposon insertion, using amplification with a transposon-specific primer, and then mapped to the genome of the organism, revealing which locations withstood transposon insertions (see Figure 1).

The data from a Tn-Seq experiment can be analyzed in several ways. First, reads can be used to ascertain the presence or absence of insertions in a gene. The probability that a gene lacking insertions is essential depends on the diversity of the transposon library (proportion of TA sites with insertions), and can be quantified using the Binomial [7], negative-Binomial distribution [8], or Extreme Value distribution [9]. Alternatively, the number of reads at each site (“read count”) can be analyzed instead of the mere presence or absence of insertions. It can be argued that the read count carries additional information because it reflects the abundance of certain clones in the library, and hence the degree to which a region of the genome is essential. Zhang et al. described a non-parametric test that quantifies the significance of the sum of read counts within a sliding window (400–600 bp) along the genome to detect essential regions [10].

Both analysis approaches have challenges, depending on the quality of the transposon library and sequencing dataset. Methods that only look at the presence or absence of insertions can be susceptible to spurious reads, such as isolated reads that map to an essential region of the genome only because they have base-call errors. However, it is difficult to set a threshold for a minimum number of reads, since other sites with a single read might be legitimate. On the other hand, methods based on read counts are susceptible to several sources of variability, including spikes in the data, where there is a massive over-representation of reads at an isolated site. The distribution of read counts is usually observed to follow a geometric distribution, but in some datasets, a few sites might have orders-of-magnitude more reads, possibly due to an artifact such as a PCR amplification bias. This could highly influence statistics based on read counts.

It should also be noted that, even in essential genes, transposon insertions are often observed to be tolerated at the extreme N- and C-terminus of the open-reading frame (ORF). Previously, ad hoc methods were used, such as excluding insertions in the N- and C-terminal 5–20% of the ORF [5]. However, both the sliding window approach [10] and the Extreme Value distribution [9] based on the length of the longest sub-sequence of TA sites without insertions are designed to be robust in spite of insertions at the termini of essential genes, and have been used to identify individual essential domains within genes [9,10].

In this paper, we describe a novel method for analyzing Tn-Seq data using Hidden Markov Models (HMMs). HMMs are useful for analyzing sequential datasets, in which a sequence of observed values is explained by an underlying state sequence (i.e. “essentiality” of each site, which is not directly observed). For example, the genome of an organism can be viewed as an alternating sequence of essential and non-essential regions. We show how an HMM can be designed to incorporate information from read counts at individual TA sites to infer the probability distribution over states, and then use the Viterbi algorithm to infer the most likely state sequence (labeling of each site as essential or non-essential). The sequential-dependence of the model (conditional probability of a state conditioned on the previous neighboring site) helps disambiguate the interpretation of each site, thereby coupling neighboring sites together. The resulting state transition model affords a 'smoothing’ of the read-count data, where, for example, TA sites with no insertions in non-essential regions (e.g. because they are absent from the library) are tolerated because neighboring sites have insertions. However, if a consecutive sequence of TA sites with no insertions is long enough, the most probable state sequence, as determined by the Viterbi algorithm, switches locally to essential, providing a different labeling of that region.

The incorporation of read-counts in this HMM requires defining appropriate likelihood functions. We use the geometric distribution to capture the conditional probability of read-counts in non-essential regions, reflecting the fact that sites with high read counts (far above average) are observed with much lower frequency than those with lower read counts. Furthermore, the transition probabilities of the HMM must be carefully defined so that the minimum length of essential regions matches our expectations. A major contribution of this paper is to show how to calibrate these parameters so that the performance of the HMM will be reasonable and robust across a range of datasets, including those with high or low insertion density (a function of the diversity of insertion library), and those with high or low mean read counts (a function of how much sequencing data is collected).

**Figure 2:**
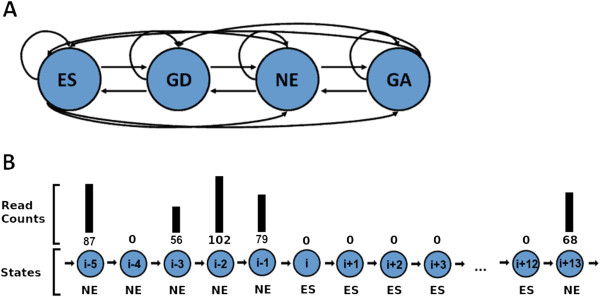
**Hidden Markov Model architecture.(A)** Diagram of the fully connected HMM structure. From left to right, the states represent read counts of increasing magnitude (essential, growth-defects, non-essential, and growth-advantage). **(B)** Diagram of the state transitions (from *q*_*i*-2_ to *q*_*i*+13_) and their corresponding emissions (i.e. read counts). A transition is made from the non-essential state to the essential state at time *i*+1, as the essential state is most likely to explain the consecutive observations of no insertions (from *q*_*i*+1_ to *q*_*i*+12_).

In addition, we extend the HMM with two extra states, one representing regions with particularly “low” read counts, and one representing regions with higher than average read-counts (see Figure 2). Genes belonging to the former class of genes have been characterized before in M. tuberculosis and referred to as “growth-defect” genes [3], as these are genes whose disruption leads to impaired growth of the organism. We continue this convention here, labeling those genes with depressed read-counts as “growth-defect” (despite the fact that these genes code for proteins whose normal function contribute to growth) to be consistent with the prior literature. Growth-defect regions are not completely devoid of insertions (as essential regions would be), but have a lower number of insertions than non-essential regions (on average), suggesting that these clones did not grow as well and had lower abundance due to competition with other clones in the library.

Similarly, the latter class of genes (i.e. those with higher than average read-counts) are labeled “growth-advantage” genes. These could represent genes that have a metabolic cost (e.g. biosynthesis of a secreted toxin) and are not necessary for growth in vitro. The addition of these two states to our HMM allows it to distinguish regions in Tn-Seq data with suppressed or unusually high read counts in a statistically rigorous way.

## Methods

The HMM in this application is defined in a straightforward way (see Rabiner for details [11]). We are given a sequence of observations, *c*_1_..*c*_*n*_, which represent read counts at each TA site throughout the genome. We assume a generative model in which the read count at each site is determined by the local state of each site, which is hidden (i.e. not directly observable). Each TA site is assumed to be in one of four states: *q*_*ES*_ (essential), *q*_*GD*_ (growth-defect), *q*_*NE*_ (non-essential), *q*_*GA*_ (growth-advantage).

From a given sequence of observations (read counts), we want to infer the most probable state sequence *q*_1_..*q*_*n*_ that could have generated it, based on the joint probability of counts and states:

(1) $$\underset{\mathrm{qi..qn}}{arg max}\;p(q_{1}\ldots q_{n},c_{1}\ldots c_{n})$$

HMMs are based on the Markov property, i.e. that observations and successor states only depend on the current state and are conditionally independent of previous history:

(2) $$p(c_{i}|q_{1},\ldots ,q_{i})=p(c_{i}|q_{i})$$

(3) $$p(q_{i+1}|q_{1},\ldots ,q_{n},c_{1},\ldots ,c_{n})=p(q_{i+1}|q_{i},c_{i+1})$$

Thus, because of this conditional independence, the total joint probability can be written as:

(4) $$p(q_{1},\ldots ,q_{n},c_{1},\ldots ,c_{n})=p(q_{1})\prod p(q_{i+1}|q_{i},c_{i})p(c_{i}|q_{i})$$

**Figure 3:**
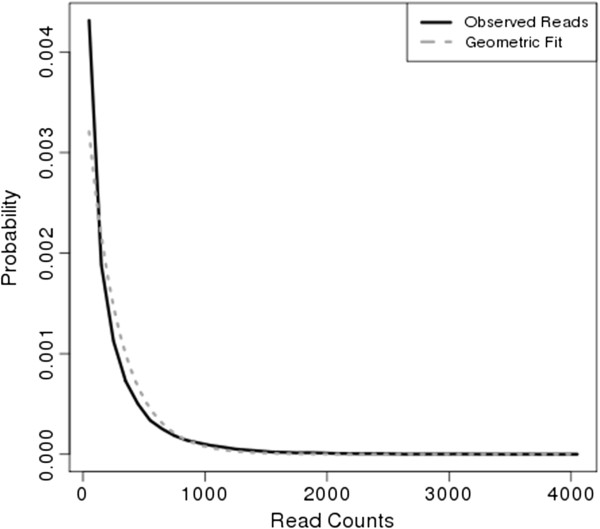
**Geometric fit of read count distribution.** Histogram of read-counts for a library of *M. tuberculosis* transposon mutants (black, solid vertical lines), fitted with a geometric distribution with parameter $\theta =1/\bar{c}$ (dashed line).

The model we propose depends critically on specifying an appropriate likelihood function for read counts. In Tn-Seq experiments, the distribution of read counts can be approximated through a geometric distribution, in that sites with lower counts are more common, and sites with high counts (far above average) are much more rare. An example histogram in shown in Figure 3 (taken from an *M. tuberculosis* H37Rv dataset [6]).

Thus we model the likelihood function (i.e. emission probability) for *q*_*NE*_ as geometric:

(5) $$p(c_{i}|q_{\text{NE}};\theta )∼{\left(1-\theta \right)}^{c_{i}}\theta$$

The function is parameterized by *θ*, which represents the Bernoulli probability of insertion for the geometric distribution. The maximum-likelihood estimate for this parameter is $\theta =1/\bar{c}$, where $\bar{c}$ is the mean read count at non empty TA sites.

**Figure 4:**
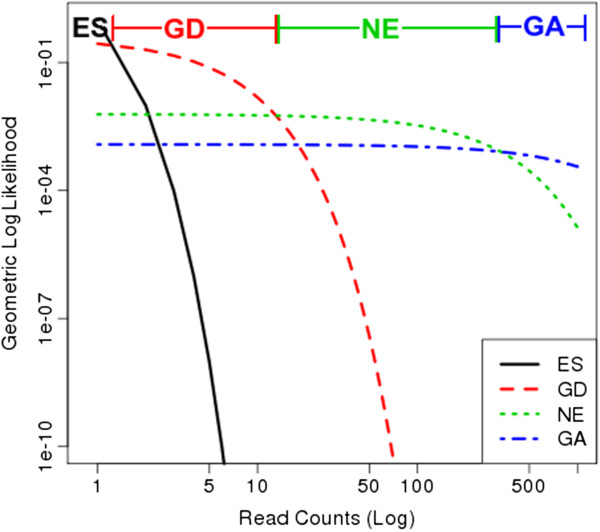
**Likelihoods.** Log-log plot of geometric likelihood functions for the essential, growth-defect, non-essential and growth-advantage states.

We also use geometric distributions as likelihood functions for the other states. For *q*_*ES*_, we set *θ* very near to 1 (e.g. 0.99), making sites with 0 counts highly probable, but also allowing sites with 1–2 reads (which could be spurious reads due to base call errors). For *q*_*GD*_ we set *θ* to be $\theta_{\text{GD}}=1/(0.01\times \bar{c}+2)$ (where $\bar{c}$ represents the mean), reflecting the fact that the growth-defect state must represent approximately ∼100× lower read counts than *q*_*NE*_ but cannot be less than 1 (converges to 2, in the limit, for very low coverage datasets). For the growth-advantage state, *q*_*GA*_, we set *θ* using five times the mean read count (i.e. $\theta_{\text{GA}}=\frac{1}{5\bar{c}}$), to capture sites with significantly more insertions (>5×) locally than what is observed on average in the genome. The net effect is that the overlapping densities of the four likelihood functions produce four distinct regions where each one dominates individually, as shown in Figure 4.

Another critical aspect of our model is the definition of the state transition probabilities, as these determine the degree of smoothing of the HMM. Let the transition matrix be defined as *T*_*ab*_=*p*(*q*_*i*+1_=*b*|*q*_*i*_=*a*). The basic assumption is that the probability of self-transition, *T*_*aa*_, should be nearly 1 for all states, while *T*_*ab*_ should be nearly 0 for *a*≠*b* (off-diagonal elements in the *T* matrix). This assumption controls the rate at which the HMM transitions from state to state, requiring a significant change in read-counts to justify a transition and smoothing over spurious reads. For simplicity, we use a fully symmetric matrix, and we allow any state to transition to any other state (i.e. we do not force sites to progress in a sequence, such as *q*_*ES*_→*q*_*GD*_→*q*_*NE*_). The magnitude of *T*_*aa*_ determines the tendency of the model to stay in one state for a certain number of steps before being forced into another state that better fits the data. This depends on several factors, including: a) the expected minimum length of essential regions (number of TA sites), and b) the relative magnitudes of the likelihood functions, which are competing to explain the read counts.

To estimate the expected minimum length of essential regions, we utilize the geometric distribution. The geometric distribution describes the probability of observing a run of successes in a row, which can be used to model the distribution of run lengths. This depends on the insertion probability in non-essential regions. Because the insertion density of the library will include essential regions with insertion probabilities which are not representative of non-essential regions. To alleviate this bias, we estimate the insertion probability, *p*_*ins*_, empirically by discarding regions with 10 or more TA sites in a row lacking insertions, and calculating the insertion density in the remaining areas. Once the insertion probability is estimated, the minimum length of essential regions, *r*^∗^, is taken to be the smallest run such that the geometric probability is less than 0.01 (i.e. *r*^∗^=argmin *P*(*r*|1-*p*_*ins*_)<0.01). Typically *r*^∗^ is in the range of 5–10 TA sites, depending on the dataset. The self-transition probability is then set as follows:

$$T_{\text{aa}}=1-{\left(\lambda_{\text{NE}}(0)\right)}^{r*}$$

where *λ*_*NE*_(0) represents the likelihood of observing a read-count of zero in a non-essential region. The rationale for this formula is that the cost of staying in a state such as *q*_*NE*_ through a region devoid of insertions, must balance the penalty incurred for observing sites with 0 read counts (*λ*_*NE*_(0)) and the number of such TA sites in a row which are likely to be observed in non-essential regions (*r*^∗^).

We will show empirically in the Results section that this adaptive method for setting the transition probabilities leads to an appropriate assignment of state labels for a variety of types of datasets, and we will examine the resulting length distribution of states produced.

Finally, given this definition of the HMM, we use the Viterbi algorithm to calculate the most probable state sequence for a given set of read counts [11]. Briefly, the Viterbi algorithm is a dynamic programming algorithm in which the probability of each state at step *i* is calculated based on the state-probability distribution from the previous step:

(6) $$p(q_{i}=a)=\max p(q_{i-1})\times p(q_{i}|q_{i-1})\times p(c_{i}|q_{i})$$

After computing this incrementally for *i*=1..*N*, a back-trace is made from the most probable terminal state $q_{n}^{*}$ to extract the sequence of states based on which states were used for updates at each step. Because the Viterbi algorithm requires the multiplication of small probabilities, and the state sequence for analyzing transposon insertions is large, an HMM may incur underflow problems. To overcome this issue, the probabilities are normalized at each iteration, as described by Rabiner et al. [11].

## Results

The HMM method was applied to a transposon mutant library of *M. tuberculosis*, constructed by Griffin et al. [6]. This library was grown on minimal media and 0.1% glycerol, and was sequenced on an Illumina GAII sequencer with a 36 bp read length, resulting in approximately 6 million reads. The reads were mapped to the H37Rv genome, and the read counts at each location in the genome were quantified (i.e. *c*_1_..*c*_*N*_). The H37Rv genome is 4,411,532 bp in length, with a GC-content of 65.6%. It contains a total of 74,605 TA sites, spaced on average 59 bp apart. The overall insertion density, defined as TA sites with at least one insertion (*c*_*i*_≥1), is 54.18% (39,762) of all possible insertion sites. The average read-count at these locations is $\bar{c}=195$ (discarding the top 5% for robustness).

The mean read count was used to calculate the *θ* parameter for the emission probabilities of the four states as described above. Using these parameters, the most likely sequence of states responsible for the observations was obtained through the Viterbi algorithm. This sequential ordering of states provides an assessment of the essentiality of the entire H37Rv genome, regardless of gene boundaries. Table 1 contains some statistics for the distribution of states and their observations.

**Table 1:** Statistics for state classifications

	**Total %**	**Mean #**	**Mean**	**Mean**
	**of genome**	**TA sites**	**insertion**	**read**
			**density**	**counts**
**Essential**	16.6	26.9	0.006	0.2
**Growth-defect**	4.1	29.2	0.20	22.5
**Non-essentials**	78.0	111.6	0.7	220.5
**Growth-advantage**	1.3	32.1	0.9	701.1

Statistics for essentials, growth-defect, non-essential and growth-advantage regions. Mean insertion density is defined the proportion of TA sites containing at least one insertion averaged across the regions belonging to a given state. Mean read counts are defined as the mean value of non-empty read counts (i.e. *c*_*i*_ > 1), averaged across all regions belonging to the given state.

**Figure 5:**
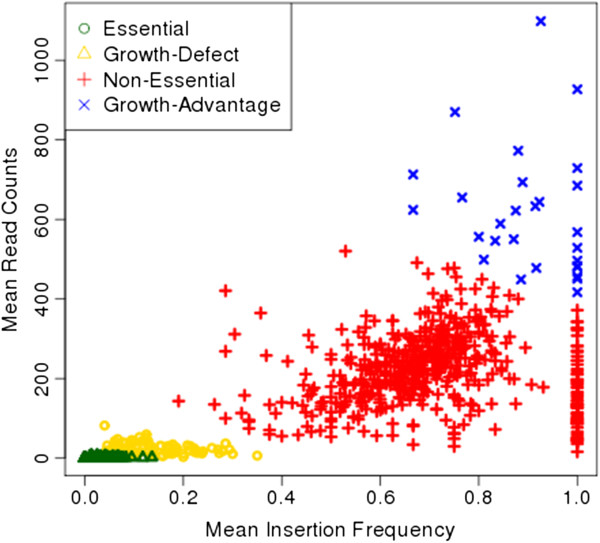
**Mean insertion density and read counts for regions.** Scatter plot of mean non-zero read counts and mean insertion frequency for the regions identified by the HMM. Regions are defined consecutive sites belonging to the same state, and are colored according to their states (Essential regions: green circles; Growth-Defect regions: yellow triangle, Non-Essential: red cross, and Growth-Advantage: blue x’s).

A total of 16.6% of the genome is labeled by the essential state (*q*_*ES*_). This is close to the expectations for bacterial organisms, where roughly 10%-15% of the genome is considered to be essential [2]. The majority of sites are labeled non-essential (78%), with a small percentage of sites labeled as growth-defect and growth-advantage (4.1% and 1.3%). Essential states averaged a very small number of insertions and read counts (0.006 and 0.2 respectively), demonstrating that the HMM is associating the essential state with stretches devoid of insertions, though these locations can occasionally contain insertions with a very small number of reads so long as as the observations at neighboring sites are consistent with essentiality. In contrast non-essential regions have a mean insertion density of 70%, and mean read counts of 220 in this dataset. Growth-defect regions have some insertions but these are dramatically reduced (20% density and a 10-fold reduction in mean read counts). Insertion density in growth-advantage regions is almost saturated (90%), and mean read counts are on average >3× larger. As can be seen in Figure 5, both the mean read counts and insertion frequencies among the states increase with the levels of non-essentiality (i.e. *q*_*ES*_→*q*_*GD*_→*q*_*NE*_→*q*_*GA*_), reflecting the fact that the HMM is successfully separating regions with average read counts and insertions from those with counts significantly lower or significantly higher than average.

**Figure 6:**
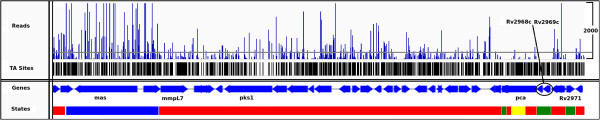
**Read counts and state classifications.** Read counts and state classifications for a 57 kb region of the H37Rv genome is shown. Essential regions are shown in green, growth-defect regions in yellow, non-essential regions in red, and growth-advantage regions in blue. Read counts are truncated at 2,000 (with a max of ∼3,000 in this region), and the mean read count in the library is represented by a gray horizontal line. Figure created using IGV - distributed by the Broad Inst. http://www.broadinstitute.org/igv/.

Figure 6 shows the read counts and state labels observed in a representative ∼57 kb region of the genome. Genes are shown as blue arrows, and the corresponding state classifications are shown at the bottom of the figure. As evident from this figure, the HMM takes into consideration the fluctuation in read counts observed. Regions devoid of insertions are classified as essential (green), those with read-counts close to the average in the library are classified as non-essential (red), while those regions with lower and higher read counts than average are classified as growth-defects (yellow) and growth-advantage (blue) respectively. Notice that *mas* (mycocerosic acid synthase, which is involved in PDIM biosynthesis) has much higher read counts than the average, and is therefore identified as a growth-advantage region. A long region of the genome is identified as non-essential as it contains read-counts that are closer to the average, despite occasional large spikes in the read-counts. This region includes *mmpL7*, which matches the expectations that most genes in the MmpL family are non-essential in vitro [12].

### Analysis of essentiality of individual genes

While the Viterbi algorithm does not take into consideration gene boundaries when determining the labeling of states, it is often necessary to determine the essentiality of individual genes in the genome. To determine individual calls of essentiality, each gene is assigned the essentiality class belonging to the most frequent state found within its boundaries. However, because genes may contain a mixture of essential and non-essential domains, genes are also classified as essential if they contain sub-sequences of sites belonging to the *q*_*ES*_ state, which are statistically longer than expected. Thus a gene is also classified as essential if it has at least *n* sites labeled as *q*_*ES*_, where *n* is 3*σ* above the expected maximum run length for the gene, based on the Extreme Value Distribution [6].

The essentiality assignments obtained through the HMM method can be validated by comparing to those obtained by Sassetti *et al* with the Transposon Site Hybridization (TraSH) method [13], which used a completely different experimental methodology for read-out (hybridization versus sequencing). This method has been used to assess the essentiality of *M. tuberculosis* in vivo and in vitro [3,14], by quantifying hybridization to DNA microarrays imprinted with representative oligos for each gene. Table 2 shows a comparison between these two methods. Due to the significantly different methodologies, a true comparison between these methods is difficult. For instance, Sassetti et al. recognized that TraSH probes for essential genes may actually hybridize to adjacent non-essential regions, particularly if the genes are small. While the HMM does not depend on hybridization, it may have a difficulty transitioning from one state to another depending on the size of the gene. In addition, libraries used by these methods were grown on different media and therefore are likely to identify genes that are involved in pathways that correspond to the specific growth media used.

**Table 2:** Comparison of essentiality predictions with TraSH

		**HMM method**				
		**Essential**	**Growth-defect**	**Non-essential**	**Growth-advantage**	**Total**
**TraSH**	**Essential**	427	54	132	0	613
	**Non-essential**	76	35	2383	20	2514
	**Total**	503	89	2515	20	3127

Comparison of essentiality predictions with the TraSH method.

Despite these limitations, there is significant agreement in their assessment of essentiality, with 89.9% of essential and non-essential genes in concordance with the previous results (70% concordance between essential genes, and 95% among non-essential genes). Approximately half of the genes labeled as 'growth-defect’ by the HMM were previously determined to be essentials, and half as non-essentials, reflecting the borderline nature of these genes and the utility of having an intermediate category. These are discussed further below. 27 genes were called 'growth-advantaged’ due to an excess of transposon insertions, and all of these were previously categorized as non-essentials.

Sassetti et al. [3] also defined a set of 42 'growth-defect’ genes. Importantly, these were not characterized by experimentally determining growth rates in individual transposon-insertion mutants. Rather, they were identified as genes that matched the criterion for 'non-essential’ on the first plating of the library (hybridization ratio >0.4, range: 0.41–2.04), but which had much lower ratios upon re-plating (hybridization ratio <0.2, thus matching the criterion for 'essential’). The interpretation of these genes is that transposon insertions were not lethal, but that the mutants had a slower growth rate, resulting in gradual depletion in the library due to competition during culturing. In the experiment from which the dataset we use was derived [6], the DNA for sequencing was extracted from the library immediately after selection, thus corresponding to the 'first plating’. Consistent with this, most of these genes (29/42) exhibited transposon insertions in our dataset and were categorized by the HMM as non-essential. We speculate that, if the library had been expanded after selection, clones with insertions in these genes would have gradually decreased in abundance.

Although the methods disagree on essentiality of some genes, some of these disagreements may be due to differences in the growth media, as well as the different interpretations of essentiality. For example *glpK*, a glycerol kinase, is necessary for glycerol metabolism (and therefore essential when grown on glycerol), but it is not necessary when the library is grown on glucose (as in the original TraSH experiment). In addition, these differences can also be due to the fact that we identify genes containing essential domains as “essential”, while this distinction was not made in the original TraSH experiments. In fact, all of the genes classified as essential by the HMM and as non-essential by the TraSH method are devoid of insertions in the majority of their TA sites or contain stretches that are significantly longer than expected, suggesting these genes are essential in this library on glycerol. Among these genes are *ppm1* (Rv2051c) and *ppp* (Rv0018c), which independent experiments have shown contain essential domains [15,16].

In addition to the TraSH method, we compare our results to those obtained with the reads-based method developed by Zhang et al. [10]. This method is capable of assessing the essentiality of the entire genome by looking at the read counts that fall within windows of 400–600 bp, and estimating a p-value for each of these windows in the genome to quantify how these regions deviate from expectations. Our results correlate well with the results obtained by window-based method, with a 93.72% match in the classification of genes (i.e. essential and growth-defects genes, as determined by our HMM, matching essential and domain-essential genes determined by the window-based method, and non-essential and growth-advantage genes matching non-essential genes). In addition, the essential and growth-defect states had TA sites with an average p-value of 0.049, and non-essential and growth-advantage states an average p-value of 0.538 (as determined by the window-based method).

### Performance on other datasets

To demonstrate that the HMM works on other datasets, we ran it on a Tn-Seq dataset from *H. influenza* (in vitro dataset SD2, [5]). The *H. influenza* KW20 genome is less than half the size of *M. tuberculosis* (1,830,138 bp, 1724 genes) but significantly more AT-rich (GC content = 38%), so there are more TA sites (131,960) but they are spaced more closely (∼14 bp apart). The Tn-Seq dataset contains 736,631 reads, hitting only 37.9% of the TA sites, with a mean read count of 11.2 (per non-zero site). Running the HMM on this lower-density dataset results in 372 genes being labeled as essential, 1150 as non-essential, 211 as growth-defect, and 6 as growth-advantage. This distribution is very close to the assignments determined by Gawronski et al. [5], who found 363 essentials (with insertions in <5*%* of TA sites in the 5–80% region of the ORF), and 211 growth-defect genes (with insertion frequencies of 5–40%). The overlap (intersection) between the essential genes detected by both their method and ours was 94% (341 genes), and the intersection between their list of growth-defect genes and ours was 60% (127).

The overlap between essential genes found by the HMM method and those found by Gawronski et al. significantly larger than the overlap between the TraSH method described above (i.e. 94% vs. 70%). This high level of agreement between the two comparisons suggests that the quality of the data used in the analysis (i.e. high-resolution sequencing data vs. hybridization ratios) contributes significantly to the quality of the analysis.

In addition, we applied the HMM method to three modified datasets, constructed to represent libraries of different sizes and different volumes of sequencing data. These datasets were constructed by modifying the original H37Rv library analyzed before, to emulate cases where transposon mutant libraries may be sparse or where the amount of sequencing performed on the library is lower (i.e. less reads).

The first dataset was constructed by setting the read counts at random TA sites to zero (i.e. *c*_*i*_=0), thus lowering the mean insertion density of the dataset while keeping the magnitude of the remaining read-counts the same. This dataset emulates libraries with significantly less diversity of insertions. The second dataset was constructed by randomly perturbing approximately one-half of the reads, lowering the magnitude of these reads while keeping the total number of insertions equal. This dataset represents libraries for which the amount of sequencing performed is significantly less, producing read counts with lower magnitudes. The final dataset was a combination of these two operations, resulting in a dataset with both lower insertion density and lower mean read count. Statistics about the distribution of reads and insertions in these datasets are shown in Table 3.

**Table 3:** Statistics for transposon mutant datasets

**Dataset**	**Insertion**	**Mean**	**Median**
	**density**	**read count**	**read count**
**Glycerol**	0.54	257	132
**Low density**	0.27	257	132
**Low reads**	0.54	76	39
**Low read & density**	0.27	76	39

Insertion density and mean read count for the glycerol, low density, low reads and, low reads and density datasets.

As can be seen in Table 4, the HMM is robust, and capable of adapting to libraries with very different insertion densities and mean read counts, providing results which are generally consistent with each other. The fraction of the genome labeled as essential is approximately the same in all four datasets (approximately 15%). Although the decreased density will result in longer stretches of the genome without a transposon insertion, the HMM is capable of adapting its parameters to become more conservative in designating regions without insertions as essential.

**Table 4:** State distribution for transposon mutant datasets

**Dataset**	**Essential (%)**	**Growth-defect (%)**	**Non-essential (%)**	**Growth-advantage (%)**
**Glycerol**	16.63	4.05	78.04	1.27
**Low density**	15.40	7.40	77.20	0.01
**Low reads**	16.13	6.78	75.51	1.58
**Low read & density**	13.75	15.64	70.55	0.06

Statistics for percentage of sites labeled as essential, growth-defect, non-essential and growth-advantage in the glycerol, low density, low reads and, low reads and density datasets.

### Growth-defect and growth-advantage genes

One of the principle advantages of our 4-state HMM is that it can distinguish local regions of the genome with significantly depressed or elevated read counts (transposon insertions). The former could represent genes whose disruption is not lethal but could lead to a growth-defect, resulting in a lower representation of clones in the library, and thus a lower abundance of sequencing reads [17]. By analogy, regions with significantly greater than average reads could represent genes whose disruption leads to a growth advantage. In the H37Rv dataset, there were 140 genes labeled as *q*_*GD*_ (growth-defect), and 27 genes labeled as *q*_*GA*_ (growth-advantage). These are discussed in turn below.

Among the genes labeled as growth-defect, there are several notable ones for which a biological explanation can be made (Table 5; see Additional file 1: Table S1 for full list). One of these is *pbpA*, a penicillin-binding protein in Mtb. Mutants have shown decreased growth rates and defective cell septation when *pbpA* is knocked out *M. smegmatis*[18]. In addition, the wild-type phenotype was restored by complementing in *pbpA* from *M. tuberculosis*, suggesting that *pbpA* plays an important role in cell-division and disruption of this gene might lead to impaired growth in *M. tuberculosis*. In fact, this region contains an average insertion density of 0.21, and an average read-count of 32, significantly below the global insertion density (0.52) and read-counts (257).

**Table 5:** Notable regions classified as growth-defect

**Orf Ids**	**Included genes**	**Insertion density**	**Average reads**	**Average nonzero reads**
Rv0015c, Rv0016c	*pknA*, *pbpA*	0.21	6.7	32.3
Rv0467	*icl*	0.23	4.0	17.6
Rv2379c	*mbtF*	0.23	3.2	14.0
Rv2380c, Rv2381c, Rv2382c	*mbtE*, *mbtD*, *mbtC*	0.29	6.4	21.7
Rv3841	*bfrB*	0.24	4.1	17.2
Rv0126	*treS*	0.26	6.6	25.8
Rv1097c, Rv1098c, Rv1099c	*fumC*, *glpX*	0.10	4.0	40.0

Notable Regions labeled as 'growth-defect’ (GD) by the HMM, in which there is a suppression of reads, suggesting an under-representation of transposon insertions in the library. Note that a lower read count is reflected in both a low read count (typically around 10–20, compared to the ∼ 250 average across the whole genome) and a low insertion density (∼ 0.20 compared to 0.54 for the whole genome).

Recent structural and enzymatic studies have shown that *bfrB* and its ortholog, *bfrA*, are not completely interchangeable. Although they are both ferritin proteins used for iron storage, *bfrB* has a 20-aa C-terminal extension that enhances its iron oxidation activity [19]. Thus growth of *bfrB* mutants might be hindered because *bfrA* cannot perform this function as efficiently. In fact, data from the original TraSH experiments shows that *bfrB* had a much lower hybridization ratio (0.73) compared to *bfrA* (2.63), suggesting clones with insertions in *bfrB* were less competitive.

Many genes in the mycobactin biosynthesis cluster (*mbtA-J*) are also labeled as growth-defect genes, suggesting that transposon mutants are viable but grow more slowly than wild-type. Because Mtb has only one (non-heme) iron acquisition system, which is mycobactin-dependent, these biosynthetic genes are essential in iron-depleted environments and non-essential in those environments that are rich in iron. Indeed, it has been shown that mycobactin-deficient mutants of Mtb, the growth rate is dependent on the iron concentration [20]. In the original TraSH experiments (plated on 7H10 medium, ∼150*μ*M Fe), *mbtB* was specifically shown to be cause a slow-growth phenotype when disrupted, with insertion mutants gradually decreasing in abundance in the library with successive platings [3].

Another interesting growth-defect gene is *glpX*. *glpK* (glycerol kinase), which is the first step in glycerol incorporation, is essential as expected (recall that this H37Rv dataset came from selection of the library on glycerol as a carbon source). *glpX* is a fructose-1,6-bisphosphatase, which also should be required when grown on gluconeogenic substrates by circumventing a non-reversible step in glycolysis pathway to generate glucose [21]. In Mtb the unexpected non-essentiality of *glpX* for growth on glycerol has been previously noted [22]. One possible explanation is that Rv2131c (*cysQ*), an inositol monophosphatase, might also have partial fructose-1,6-bisphosphatase activity [23].

*icl* (isocitrate lyase) is also identified as a growth-defect gene in this dataset. This is one of the two enzymes on the glyoxylate shunt, which has been shown to be critical for infection, based on attenuation of knockouts in mice [24]. As anticipated, *icl* is essential for growth on fatty-acid substrates like acetate [24]. However, recent evidence suggests that the glyoxylate shunt might play a role even in growth on other carbon sources such as carbohydrates. For instance, *icl* knockouts have displayed a growth-defect (2–4 day lag compared to wild-type) on glucose [25]. More recently, it has been shown that inhibitors of malate synthase (GlcB, the other enzyme of the glyxolate shunt) are active against cultures whether grown on acetate or glucose [26]. Thus, the fact that the HMM labels *icl* as a growth-defect region in this dataset obtained from growth on glycerol is consistent with these findings and suggests that *icl* plays an unexpected metabolic role in Mtb even when growing on carbon sources other than fatty acids.

Another gene identified as belonging to the growth-defect category is *treS*, which is involved in the trehalose pathway. Trehalose is one of the principle carbohydrates synthesized in mycobacteria. It is used in producing cell-wall glycolipid components (e.g. TMM and TDM, trehalose mono- and di-mycolates), and is inter-converted with other sugars like glucose and maltose. The latter are polymerized into intracellular glycogen (for energy storage) and capsular glucan. Several genes in this network have been shown to be essential in vitro, including *galU*, *glgA*, *glgB*, *pep2*, and *glgE* (all essential in our dataset). However, *treS* is labeled as a growth-defect gene. *treS* is responsible for interconverting trehalose and maltose [27,28]. It is possible that the organism is sensitive to perturbations of this network (given the essentiality of nearby genes like *glgA*, and toxicity of intermediate metabolites like maltose-1-phosphate [29]). In fact, it was previously shown that transposon-insertion mutants of *treS*/Rv0126 display a slow-growth phenotype [3].

As noted before, our 4-state HMM is also capable of detecting regions with unexpectedly high read-counts that might confer growth-advantages to the organism when disrupted. The 10 most notable growth-advantage regions are shown in Table 6 (full list is shown in Additional file 2: Table S2). One region of the genome that stands out is the PDIM locus, Rv2930-Rv2939. This locus contains genes involved in the biosynthesis of phthiocerol dimycocerosate (PDIM), including *fadD26* and *ppsABCDE*. In addition, other genes outside this locus believed to be involved in PDIM biosynthesis, like *papA5* and *mas*, are identified as well. These genes contain read counts well above the global average (∼250). *fadD26* itself has a mean read count of 818, more than three times the average throughout the genome. *ppsDE* had a mean read count of 732, and *ppsABC* a mean read count of 463. PDIM is a cell-wall associated glycolipid that modulates the immune response in the host [30,31]. Although it is required for virulence (as strains with disruptions of these genes are attenuated in animal models [32]), it is not required for survival in vitro [3,5,6]. In fact, biosynthesis of PDIM requires resources and imparts a metabolic cost, hence disruption of this pathway is advantageous to cells. Due to the increased metabolic cost, it is widely observed that *M. tuberculosis* stocks maintained in the lab frequently lose the ability to synthesize PDIM via acquisitions of mutations in these genes, often leading larger colony sizes [33]. This growth advantage and consequent selection effect likely explains why clones with transposon insertions in the PDIM locus are over-represented in the library.

**Table 6:** Notable regions classified as growth-advantage

**Orf Ids**	**Included genes**	**Length of growth-advantage region**	**Average reads**
Rv3295, Rv3296	*lhr*	27	1098
Rv2939, Rv2940c, Rv2941	*papA5*, *mas*, *fadD28*	149	870
Rv2411c	-	25	773
Rv0483	*lprQ*	36	694
Rv2930, Rv2931, Rv2932, Rv2933, Rv2934, Rv2935	*fadD26*, *ppsA*, *ppsB*, *ppsC*, *ppsD*, *ppsE*	398	655
Rv1843c, Rv1844c	*guaB1*, *gnd1*	47	633
Rv0554	*bpoC*	16	622
Rv0479c, Rv0480c, Rv0481c	-	32	589

Regions labeled as 'growth-advantage’ (GA) by the HMM, in which there is an excess of reads, suggesting an over-representation of transposon insertions.

## Discussion and conclusions

The HMM described in this work enables the characterization of essentiality throughout an entire bacterial genome from sequencing data of transposon mutagenesis experiments. Although several computational methods have previously been proposed for analyzing Tn-Seq data, including some based on presence/absence of reads [7-9] as well as non-parametric models that take quantitative read counts into consideration [10], an HMM provides several advantages over these methods. For example, an HMM provides a smoothing over adjacent sites that couples them together to help disambiguate the interpretation of read counts at individuals sites. Another advantage of using the HMM is that it is not restricted to annotated gene boundaries, and can identify independent regulatory regions, non-coding RNAs, and protein domains that are required for survival. While methods that depend on a sliding-window (as developed by Zhang et al.) are also capable of assessing essentiality over the entire genome by locally averaging over adjacent TA sites, an HMM formalizes this process in a statistically rigorous way. In addition, by assessing essentiality among regions in the genome, the HMM can also tolerate insertions in the N- and C- termini of genes, without the need of discarding insertions at these locations in an ad-hoc manner as some methods have done previously [5].

A potential limitation of our method is that it does not take into consideration the doubling-rate or expansion time of the library when estimating the parameters of the model. This can affect the under- and over-representation of mutants, and therefore the number of reads these genes will contain in the sequence data. Because the HMM depends on the read counts for individual genes, it may be susceptible to libraries that are constructed in different ways. Indeed, the ability to detect essential genomic regions from transposon-insertion sequencing data is highly dependent on the quality of the dataset. In practice, Tn-Seq datasets can span a range from hundreds of thousands to millions of reads, but below some point, there are not enough reads to discriminate essential regions confidently. Similarly, well-saturated libraries can have insertions at > 50*%* of TA sites, but other datasets are more sparse (from less diverse libraries), again increasing the difficulty in distinguishing essential regions. Additionally, some sites may contain reads that are orders-of-magnitude larger due to PCR amplification or the development of “hotspots” due to the interactions of the transposon and the organism’s replication machinery [34]. These problems could be alleviated by comparing cultures before and after the passage in the media or comparing datasets derived using different transposons with specificity to different sites. While not directly filtered by our HMM, we showed that the parameter estimation equations we propose work on a wide range of real datasets. In particular we show that they work on dense datasets (*M. tuberculosis*[6], 54% insertion density), as well as sparse ones (*H. influenza*[5], 38%), and even on artificial datasets down to 25% insertion density. In all cases, the HMM is stable in that it outputs about the same proportion of essential regions, so as the volume of data decreases, the HMM adapts its predictions and becomes increasingly conservative.

One of the major advantages of using an HMM to analyze transposon mutagenesis data is that additional states may be introduced to capture distinct types of genomic regions (beyond essential and non-essential). In this work we add states to capture regions whose disruption leads to a growth defect or a growth advantage. van Opijnen et al. [17] have shown that relative abundance of insertions in a gene can be correlated quantitatively with growth rate (doubling time), though it depends on the number of generations the culture is allowed to grow. Eventually, clones with insertions in growth-defect genes will be depleted from the library due to competition (exponential growth). Sassetti et al. found substantial reductions in abundance, even after a second round of plating, where hybridization ratios for certain genes dropped from non-essential (>0.40) to essential (<0.20). While our model only distinguishes one class of growth-defect genes, it could be expanded to more states, discerning finer gradations of growth impairment [17].

A total of 140 growth-defect genes, and 27 growth-advantage genes in *M. tuberculosis* H37Rv were identified by our 4-state HMM, several of which have been shown to be biologically valid, as knockout mutants have been shown to grow slower (or faster) than the parental strain. Identifying these finer distinctions of essentiality (in addition to the traditional essential and non-essential categories) can enrich our understanding of the biological roles of genes. For example, we found that *icl* (isocitrate lyase) is labeled as growth-defect (on glycerol). Historically, ICL has been viewed as essential in *M. tuberculosis* specifically for growth on fatty-acid substrates, and non-essential otherwise. However, that view is too limiting. *icl* knock-out strains have in fact been observed to display a growth defect when grown on glucose [25], and the suppression of reads we observed in *icl* in the transposon mutagenesis data is consistent with this (glycerol being another carbohydrate-like substrate), suggesting that this gene plays an additional role that is not well-appreciated.

## Availability and requirements

The HMM model utilized in this paper is made available online in the **Tn-HMM** package.

**Project name:** Tn-HMM

**Project home page:**http://saclab.tamu.edu/essentiality/HMM/

**Operating system(s):** Linux, Windows

**Programming language:** Python

**License:** Creative Commons Attribution-NonCommercial

**Any restrictions to use by non-academics:** None

## Competing interests

The authors declare that they have no competing interests.

## Authors’ contributions

TRI and MAD were jointly responsible for the development of the algorithm and the writing of the manuscript. Both authors read and approved the final manuscript.

## Supplementary Material

Additional file 1 **Table S1.** Excel spreadsheet (table_S1.xls) containing a list of growth-defect regions identified by the HMM in *M. tuberculosis* H37Rv grown on glycerol (data from Griffin et al. 2011). Click here for file

Additional file 2 **Table S2.** Excel spreadsheet (table_S2.xls) containing a list of growth-advantage regions identified by the HMM in *M. tuberculosis* H37Rv grown on glycerol (data from Griffin et al. 2011). Click here for file
